# Preferential sites for intramolecular glucosepane cross-link formation in type I collagen: A thermodynamic study

**DOI:** 10.1016/j.matbio.2015.06.001

**Published:** 2015-10

**Authors:** Thomas A. Collier, Anthony Nash, Helen L. Birch, Nora H. de Leeuw

**Affiliations:** 1 Department of Chemistry, University College London, 20 Gordon Street, London, WC1H 0AJ, United Kingdom; 2 Institute of Orthopaedics and Musculoskeletal Science, UCL, RNOH Stanmore Campus, London, United Kingdom

**Keywords:** ECM, extracellular matrix, MD, molecular dynamics, AGEs, advanced glycation end products, SLRP, small leucine-rich proteoglycan, HS, heparan sulphate, HSP-47, heat shock protein 47, MMP1, Matrix Metalloproteinase-1, DS, dermatan sulphate, MIDAS, metal ion-dependent adhesion site, IL-2, interleukin 2, Collagen, Glycation, Molecular dynamics, Protein cross-linking, Glucosepane, Advanced glycation end products

## Abstract

The extracellular matrix (ECM) undergoes progressive age-related stiffening and loss of proteolytic digestibility due to an increase in concentration of advanced glycation end products (AGEs). The most abundant AGE, glucosepane, accumulates in collagen with concentrations over 100 times greater than all other AGEs. Detrimental collagen stiffening properties are believed to play a significant role in several age-related diseases such as osteoporosis and cardiovascular disease. Currently little is known of the potential location of covalently cross-linked glucosepane formation within collagen molecules; neither are there reports on how the respective cross-link sites affect the physical and biochemical properties of collagen. Using fully atomistic molecular dynamics simulations (MD) we have identified six sites where the formation of a covalent intra-molecular glucosepane cross-link within a single collagen molecule in a fibrillar environment is energetically favourable. Identification of these favourable sites enables us to align collagen cross-linking with experimentally observed changes to the ECM. For example, formation of glucosepane was found to be energetically favourable within close proximity of the Matrix Metalloproteinase-1 (MMP1) binding site, which could potentially disrupt collagen degradation.

## Highlights

• We conduct fully atomistic molecular dynamics simulation of fibrillar collagen.
• Glucosepane cross-link formation is energetically favourable at six positions.
• Positions identified are within key collagen biomolecule sites.
• Positioning of sites may have a significant effect on tissue function and integrity.

## Introduction

Collagen plays an important structural role in the extracellular matrix (ECM) of all vertebrates and accounts for over a quarter of the dry mass of the human body [1], [2]. Collagen forms a family of proteins [3], but the fibril-forming type I collagen dominates in organs and tissues that require tensile strength, such as tendon, ligament, bone and the dermis. The precise way in which type I collagen molecules are organised and chemically linked into collagen fibrils provides these tissues with the specific mechanical properties required for efficient biological function. During ageing, however, the skeletal and other connective tissues show a gradual decline in their ability to function effectively and the incidence of injury and disease increases [4]. These changes occur, in part, due to adventitious chemical modifications to collagen over time, such as the addition of sugars (glycation) and the formation of advanced glycation end products (AGEs) [5].

**Fig. 1:**
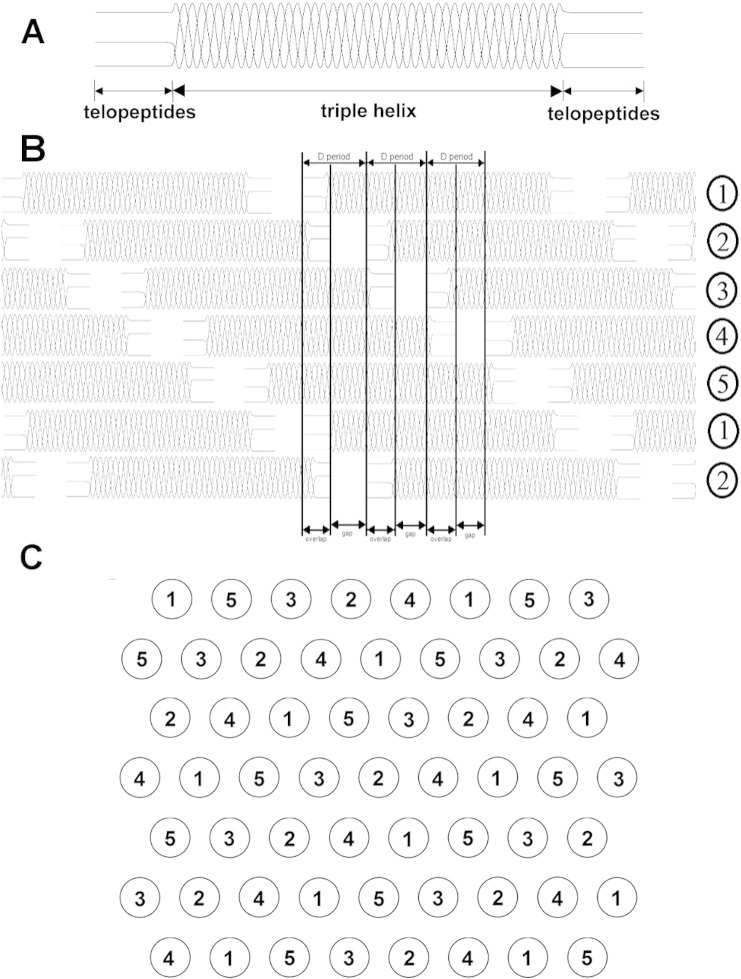
Schematic of (A) a single collagen protein; (B) and (C) are schematics showing the supramolecular arrangement of the collagen molecules in a collagen fibril. Specifically (B) shows the staggered axial alignment in the fibril, with each collagen molecule represented as a straight rod. (C) Cross section through a fibril in the overlap region with each collagen molecule represented as a circle. In both (B) and (C) the numbers represent the five possible axial alignments of the proteins.

Collagen molecules, are 300 nm long rope-like macromolecules comprising three polypeptide strands twisted into a continuous 1.5 nm diameter right-handed triple helix, terminated at both ends by short non-helical telopeptides. According to the Hodge–Pertruska model, the collagen molecules line up in a parallel staggered side-by-side arrangement to form hydrated fibrils, shown in Fig. 1 [6]. These collagen fibrils have varying lengths and diameters dependent on the organism and location of the tissue. Collagen fibrils within tendon typically have diameters ranging between 20 and 150 nm and a length on the millimetre scale. For example in human Achilles tendon average fibril diameters of 50–90 nm have been measured whereas in the flexors and extensors of the fingers diameters are 20–60 nm [7]. The collagen molecules within a fibril are stabilised by the formation of enzyme-mediated cross-links within the telopeptide region of the molecules soon after the fibril is formed [8]. Collagen arranged and cross-linked in this way results in tissues with high tensile strength. In addition to a mechanical contribution, the precise arrangement of collagen molecules within the fibril governs important interactions with other matrix macromolecules and the cellular component of the tissue. For example, decorin, a small leucine-rich proteoglycan (SLRP), binds to fibrillar collagen at specific sites, where sufficient space is available to accommodate the protein core [9]. The binding of decorin plays a role in regulating the collagen fibril diameter by inhibiting lateral growth of the fibril [10]. Collagen also contains cell interaction domains, which are able to bind to integrins on the cell surface. This cell matrix interaction is important for mechanotransduction and other cell signalling events.

Whilst enzyme-mediated cross-linking is physiological and provides functionality to tissues, glycation-mediated cross-linking is considered to be pathological and is thought to jeopardize the functionality of the musculoskeletal system [11], [12]. Glycation cross-links are formed by a series of reactions initiated by a reactive dicarbonyl metabolite attack on the free amine group of lysine residues. The end-products can link polypeptide chains together within or between collagen molecules giving rise to AGE cross-links. As a long-lived protein collagen is particularly vulnerable to AGE cross-linking. For example, in tendon a half-life of up to 200 years was calculated for the collagenous component based on aspartic acid racemization [13]. Evidence suggests that mechanical and biological properties of collagen are disturbed by age-related chemical modifications. A previous study by Reddy et al., found that in vitro incubation of rabbit Achilles tendon in ribose increased levels of the AGE pentosidine and increases Young's modulus by 159% from 24.89 ± 1.52 MPa to 65.087 ± 14.41 MPa, suggesting that the presence of AGE cross-links increased stiffness of soft tissue [14]. Other studies have shown that glycation weakens collagen proteoglycan interaction and results in impaired cell migration [15].

Although a series of AGE cross-links is possible, the lysine–arginine–glucose AGE cross-link glucosepane is the most abundant in collagen with levels 100 to 1000 times higher than all other currently known cross-links [16]. The concentration of AGE cross-links are found to vary depending on the tissue and AGE type, with glucosepane found in concentrations of 250 pmol/mg whereas MODIC and DOGDIC are found in much lower quantities, < 75 pmol/mg and < 5 pmol/mg, respectively [11]. One theory to explain the abundance of glucosepane, is that the final carbonyl rearrangement from the Amadori product undergoes a non-reversible dehydration step which ultimately leads to an accumulation of glucosepane, whereas AGEs formed by other glycation agents are formed reversibly [17]. The concentration of glucosepane in tissue is dictated primarily by two factors: the concentration of glucose; and the rate of turnover of collagen. For example, the hyperglycemic environment of diabetic patients leads to higher levels of AGEs in human collagen skin samples and glomerular basement membrane collagen samples compared to non-diabetic patients [11], [18]. In addition short-lived collagen molecules such as those found in the skin have a lower glucosepane concentration than collagen molecules in articular cartilage, which has a much longer lifespan [19].

Although the absolute levels of AGE cross-links are important, the exact position within the collagen molecules are critical in determining the impact that they have on the collagen properties. To date, little work on the identification of residues involved in inter- and intra-molecular glucosepane cross-linking had been reported. A previous study identified potential sites from a 5 Å cut-off between the terminal nitrogen atoms N^ε^ and N^ζ^ for lysine and arginine respectively, during a 100 ns molecular dynamics (MD) simulation [20]. That particular study did not, however, take into account whether the identified sites were thermodynamically favourable, which will be a critical factor in determining the likelihood of glucosepane formation. In this study we have used a fully atomistic model of an entire collagen molecule in a fibrillar environment to identify, based on energetics, the residues responsible for forming intra-molecular glucosepane cross-links. We discuss how the identified potential sites of glucosepane formation might cause disruption of the biological and mechanical properties of collagen.

## Results

A distance-based criterion search is used within a model collagen molecule to identify lysine and arginine residues within 5 Å of one another. The cut-off was chosen based on two main factors. Firstly, previous studies have suggested that both residues, within 5 Å, are a strong indicator for preferential glycation [21], and secondly, doubling the distance between the nitrogen atoms within glucosepane (approximately 2.5 Å and 3.7 Å) could reveal a reasonable number of potential sites where cross-links are likely to form [21]. Under physiological conditions and exploiting the D-band periodicity to replicate the dense fibrillar environment we performed a set of atomistic MD simulations. Each system set-up comprised of a collagen molecule and a single intra-molecular cross-link at anyone of the identified lysine–arginine sites. We identify whether a site is a likely candidate for glucosepane formation if the total energy of the collagen molecule is lower in the presence of a bound glucosepane cross-link compared to an unbound glucose molecule. Finally, using the candidate cell and matrix interaction domain map of the collagen fibril produced by Sweeney et al. [22], we identify collagen-dependent biological processes native to the ECM which coincide with the location of the cross-link sites.

A distance-based criterion search identified 24 potential glucosepane cross-link sites distributed along the length of the triple helical portion of the collagen molecule. The distribution and position of the sites over the length of the collagen molecule is illustrated in Fig. 3, with exothermic binding enthalpies shown in green and endothermic binding enthalpy sites in red.

The average binding enthalpies of the glucosepane cross-links were calculated using the total energy over the last 25 ns from each cross-linked collagen simulation, where the average total energy of the native reference collagen model was subtracted from the total energy over the last 25 ns for the cross-linked collagen. The binding enthalpies are reported in Table 1. Residue numbering originates from the original Uniprot entries. The statistical error of the formation enthalpy was calculated using the standard error of the mean, which was found to be approximately 0.7 kcal mol^− 1^ for all of the cross-linked simulations. Sites 1 and 23 were found to have strong steric clashes with neighbouring images of the collagen molecule. It was therefore decided early on that these cross-links would not form within the fibrillar environment, and hence the simulations were not continued.

Six potential sites yielded an exothermic enthalpy change on formation of the glucosepane cross-link between the two identified amino acids, all of which occur within regions which are thought to be of biological significance. The details of the biomolecule binding site overlaps for each favourable cross-link site are presented in Table 2. Specifically, site 2 occurs at a position local to the interaction sites of heparan sulphate (HS), α1α1 and α2β1 integrins, and an enzyme mediated cross-link [22], [23], [24], [25]. Site 7 is within the binding sites of heat shock protein 47 (HSP-47) chaperone and the proteoglycan decorin [22], [26]. Site 13 was found within the binding region of phosphoryn, a protein of dentine which plays a role in bone mineralization [27]. Site 17 occurs within the binding sites of α2β1 integrin, HSP-47 chaperone, a fibrillogenesis inhibitor, and is also within close proximity of the binding site of the collagenase Matrix Metalloproteinase-1 (MMP1) [26], [28]. Site 20 is within the binding site of dermatan sulphate (DS) proteoglycan and the secreted protein factor interleukin 2 (IL-2) [22], [29], [30]. Finally, site 22 is also within the IL-2 binding domain as well as the binding location of the anticoagulant heparin [25], [30]. The remaining 18 sites were found to have energetically unfavourable changes in enthalpy upon cross-link formation.

## Discussion

It has long been assumed that the presence of AGEs in and between collagen molecules alters their physical properties, as well as increasing the lifetime of collagen molecules within the body.

In our study we have identified six favourable cross-link sites, which are presented in Fig. 4 along with the neighbouring amino acids. After cross-link formation at these sites, no significant deviation to the backbone was observed relative to their corresponding position in the native model. Therefore, it can be inferred that the major contribution to the decrease in enthalpy is an increase in the number of favourable side-chain to side-chain interactions or side-chain to backbone interactions, in addition to the contribution of glucosepane to protein interactions. Here we give an example using the average bond distances of these additional interactions found after cross-link formation. A side-chain to side-chain interaction found within the immediate vicinity of position 20, is a potential hydrogen bonding interaction between ^983^Asn (α2) HD22 and ^1055^Arg (α1a) HE with an average bond length of 2.16 Å, over the last 25 ns of the simulation. Most of the additional interactions however occurred between side chains and the backbone of the polypeptide. For example, after cross-linking at position 17 an additional hydrogen bonding interaction is formed with an average length of 2.00 Å between ^956^Arg (α1b) HE and the backbone carbonyl of ^957^Gly (α1b) O. The formation of a cross-link results in four additional carbon containing covalent bonds being present in the system, thus lowering the energy of the system. However, the overall enthalpy change between the bound and native models is a combination of the energy released by the formation of the covalent bonds plus the additional energy contributions from the increased number of interactions, both favourable and unfavourable. In addition, cross-links potentially contribute additional electrostatic interactions with the surrounding residues. One example is the formation of two long-range electrostatic interactions with an average distance of 3.05 Å and 3.22 Å, between the cross-link hydroxyl groups and the carbonyl oxygen of ^679^Gly (α2) O at position 13.

Eighteen of the potential cross-linking sites identified on the distance based criteria, were found to be energetically unfavourable. There are three main reasons for the unfavourable formation enthalpies of these binding sites: the local structure of the collagen at the site; the configuration of the binding side-chains; and the presence of steric clashes and close contacts. Close contact can occur between residues within the same collagen molecule, as is the case at site 19 where the arginine N^η^ is within 3.5–5.0 Å of the O^δ^ of the neighbouring ^1024^Asp residue, or between residues on neighbouring collagen molecules within the fibril, e.g. at site 15 where there is a large number of close contacts of 1.5–4.0 Å between several positions on ^851^Lys and ^1085^Lys on a neighbouring molecule. When a cross-link forms, a rotation of both side chains around the α-carbons may be necessary, which causes the residues to adopt configurations not experienced in the native state. Upon cross-linking the ^851^Lys's movement is severely restricted and thus the presence of the nearby ^1085^Lys residue, which also has its movements limited by nearby residues from neighbouring molecules, causes an increase in energy as the two residues come into close contact. Smaller contributions to the energy result from the local configuration of the linking side-chains, which arises when upon cross-link formation the number of degrees of freedom for the bound residues decrease. For example, in site 8 we see a rotation of 180° around the C^β^–C^γ^, thus adopting an eclipsed conformation with both the two larger groups located on the same position on their respective carbon atoms. The local structure of the collagen around the binding site dictates the extent to which the two previously described contributors to the energy occur, as the proximity and size of residues near the site can have a large impact on the ability of the residue to form a glucosepane cross-link.

Gautieri et al. [20], identified potential sites on a homology model of the human sequence based upon the amount of time the two potential cross-linking side chains were within 5 Å of one another. Our study builds on this work, by conducting fully atomistic MD simulations of the actual cross-linked molecules to ascertain whether the structural rearrangement around the binding site is energetically favourable or not. Direct comparison between the sites identified in our study and those of the previous work is not possible. The local structure will vary due to the different methods used to incorporate the human sequence and the fact that the explicit cross-links in our study influence the local environment.

The presence of glucosepane cross-links at any of the six identified sites in this study could result in an alteration in the physical properties of the collagen molecule. In addition, glycation of side chains in, or close to, binding sites for ECM biological molecules, such as heparin, proteoglycans or collagenases will prevent collagen protein interactions and impede essential ECM functions. Sweeney et al., created a descriptive map of human type I collagen with marked ECM interaction domains based on an earlier map and database [22], [31].

The amino acid sequence and structural information used in this study was that of *Rattus norvegicus* owing to the availability of the experimental X-ray diffraction data and the strong biosimilarity between rat and human sequences. Sweeney et al., used the same crystallographic data to obtain a descriptive map and then localised functional domains of the human type I collagen onto the rat type I microfibril [22]. By using a similar approach we have been able to directly map favourable and unfavourable cross-link sites onto the human sequence and compile a list of ECM interaction sites that may be impeded by cross-linking of collagen. Our modelling approach adopts a homotypic microfibril of type I collagen. Typically, healthy tissue however is heterotypic with tendon fibrils other minor collagen types such as type III and type V. Given that our study focuses on intramolecular cross-links, a heterotypic microfibril composition would have little effect if any on the predicted cross-links locations.

An important part of forming mechanically competent collagen fibrils is the formation of chemical cross-links between collagen molecules. These cross-links occur during fibrillogenesis and are mediated by the enzyme lysyl oxidase. This enzymatic mediated cross-linking is thought to occur from a young age between α1-^254^Lys [16], [23] and a lysine residue of a neighbouring molecule. The α1-^254^Lys residues already involved in enzymatic cross-links are therefore unlikely to be available for glucosepane cross-linking, however the α1-^254^Lys not involved in the enzymatic cross-link is available for glycation. Another active enzyme in the ECM is lysyl hydroxylase which hydroxylates lysine residues producing hydroxylysine during post-translational modification of the collagen molecule. The hydroxylysine residues within our model were included in the distance based criterion search owing to the availability of their amine group which is needed to form the glucosepane cross-link [32].

A number of biomolecular interactions identified would remain unaffected by glucosepane owing to the fact that the process in which they are involved occurs prior to secretion of the procollagen into the ECM. One particular example of this is the HSP-47 binding site, which overlaps potential cross-linking sites 2, 7 and 17. HSP-47 is, however, an intracellular collagen-stress binding protein local to the endoplasmic reticulum and responsible for maturation of a number of types of collagen.

The energetically favoured cross-link site 13 may result in structural variation of the binding site of phosphophoryn. The binding of type I collagen to phosphophoryn, the major non-collagenous dentin protein, is believed to play an important part in the nucleation of the mineral phase within the dentin matrix [27]. Phosphophoryn has a large number of Asp–Ser–Ser repeats interspersed throughout the molecule and Ser–Asp domains towards the C-terminal, both of which are readily phosphorylated [33]. In cases where most of the available Asp–Ser–Ser and Ser–Asp motifs are phosphorylated, the molecule will have a strong negative charge and thus acts as a sink for binding calcium ions, potentially directing the location and moderating the speed of mineralisation within the dentin matrix [34]. The effect of glucosepane formation on phosphophoryn binding and thus dentin mineralisation is dependent on which of the two processes will occur first. The exact point in time at which mineralisation occurs is still unknown, although it is often suggested that biomineralisation will occur shortly after fibrillogenesis [35]. If this were the case then we would not expect a glucosepane cross-link to form at this site.

Glycosaminoglycans constitute a considerable fraction of the glycoconjugates found in the ECM of virtually all mammalian tissues, where they play a significant role in the biological function of the tissue. HS and DS serve as: key biological response modifiers by acting as stabilisers, cofactors, and co-receptors for growth factors and cytokines. HS and DS also act as regulators of enzyme activity; signalling molecules in response to cellular damage, such as wounding, infection, and carcinogenesis and targets for bacterial, viral, and parasitic virulence factors for attachment and invasion. Keratan sulphate acts as a hydration agent due to its distinct water binding properties, although its anti-adhesive character has also been suggested to play a role in cell migration. Reigle et al., had observed that keratan sulphate proteoglycans and heparin experience a reduced affinity for glycated collagen, whereas DS proteoglycans exhibit no change in affinity [15]. Co-electrophoresis analysis of heparin's affinity for collagen revealed that non-glycated collagen had an appreciably stronger heparin binding (K_d_ of 100 nM) compared to glycated collagen (K_d_ of 250 nM). The same study found that the defective heparin binding found in the glycated collagen is independent of the supramolecular state of the collagen. It is possible that *intra*-molecular glucosepane cross-link formation at site 22 could reduce the concentration of bound heparin. HS is a structural analog of heparin which is therefore likely to have similar binding mechanism to the collagen, therefore we could also expect disruption to HS binding at position 2 to be disrupted by the presence of a glucosepane cross-link. This is in agreement with the work of Reigle et al., which showed that the HS proteoglycans also had a reduced affinity for glycated collagen. The blocking of heparin from binding due to the presence of glucosepane, could happen in two ways, firstly, through steric blocking of the binding site, and secondly, by altering the electrostatic potential after the occupation of the lysine side chain during cross-linking. Reigle et al., found no reduction in affinity between DS and glycated collagens, which they attributed to heparin and keratan sulphate proteoglycans having significant electrostatic contributions towards binding, unlike other proteoglycans [15]. A combination of a reduced electrostatic contribution to binding, in addition to only the arginine of the pair involved in the cross-link situated within the DS binding region at site 20, suggests that the presence of an AGE in this region would have little effect on the binding of DS. However additional investigation would be necessary to develop this suggestion further, for example through explicit modelling of the molecules and interactions.

The two major membrane-bound integrins α1β1 and α2β1 are in part responsible for eukaryotic cell-collagen (type I) interaction within the ECM. No favourable cross-linking site is present in the key collagen–cell interaction domain, however sites 2 and 17 occur at positions aligned with the cell interaction domain of a neighbouring collagen molecule. Integrins bind using their A-like domain, which contains a trench centred onto a metal ion-dependent adhesion site (MIDAS), in the presence of Mg^2 +^ or Mn^2 +^. Upon binding, a glutamine residue becomes attracted to the metal ion in the MIDAS site [36], [37]. Due to the size of the A-like domain there are some additional interactions between the integrin and residues in the neighbouring collagen molecules. The introduction of a glucosepane cross-link into the collagen cross-link sites 2 and 17 therefore alters the polarity and structure of the additional interaction sites, potentially leading to a lower binding affinity. If that were the case, one might expect to observe reduced integrin interactions.

Type I collagen acts as an extracellular store for bioactive interleukin 2 (IL-2) through reversible binding, thereby increasing the bioavailability of IL-2 in a spatial pattern dictated by the organisation of the collagen molecules [30]. The binding of IL-2 has been shown to be very site-specific, with a K_d_ of approximately 10^− 2^–10^− 8^ M and molar ratios of four to six IL-2 to one collagen molecule. IL-2 is an important stimulator and modulator of T-cell activation, adopting a key role in the pathophysiology of various immune-mediated diseases such as rheumatoid arthritis, multiple sclerosis and transplant rejection. Somasundaram et al., found that the binding of IL-2 was very sensitive to the collagen sequence and structure of the binding site, although the exact mechanism of attachment is unknown [30]. The presence of a glucosepane cross-link at either of the two favourable formation sites 20 and 22 within an IL-2 binding region may therefore have a significant effect on IL-2 binding, potentially leading to disruption in the attachment of IL-2 to the collagen, which could decrease T-cell response time.

Another important implication of the findings presented here is the fact that favourable formation site 17 has a lysine residue that is only two residues down from the proposed MMP1 binding site [22], [28]. Inhibition of the collagenase via collagen cross-linking may significantly affect the digestibility of the cross-linked tissue [38]. Although the exact mechanism of action of the MMP1 enzyme has so far not been determined in significant detail, it is known that it operates by first uncoiling the three polypeptide chains before cleaving the peptide bond. Cross-links between polypeptide chains hinder this uncoiling process [28]. However, it has been hypothesised that the MMP1 preferentially interacts with the α2(I) chain, which could mean that the presence of the cross-link would not completely hinder the uncoiling of the polypeptide chains. Yet without an identified mechanism for the binding of MMP1 and the uncoiling of the collagen substrate it is difficult to know to what extent the presence of glucosepane affects proteolysis.

In summary our study has identified six energetically favourable sites for intramolecular glucosepane formation in type I collagen. The position of these cross-links is likely to have a significant impact on collagen properties. The formation of glucosepane at these sites may explain age related changes in the functionality of type I collagen rich tissues such as tendon, ligament, bone and dermis.

## Methods

An all-atom model of a *R. norvegicus* type I collagen molecule, exploiting periodic boundary conditions to replicate the fibrillar environment, was used to study the energetics of glucosepane cross-link formation.

### Constructing the model

Our model uses the amino acid sequence for *R. norvegicus* owing to the availability of the crystal structure and similarity to the *Homo sapiens* sequence, making it suitable for this study. The methodology used to obtain a low energy conformer of type I collagen is fully described in our previous work [39], but here we provide a brief summary for clarity. A straight-chained structure of a collagen molecule with the correct helical propensity was generated using the Triple Helical Building Script (THeBuScr) [40]. The primary sequences of the collagen peptide chains α1 and α2, translated from the genes COL1A1(P02454) and COL1A2 (P02466) [41], were the inputs. The primary sequences used included the post-translational modified residues such as hydroxyproline and hydroxylysine, which were present in the UniProt entries. A custom script was used to combine the output from THeBuScr and the fibrillar arrangement taken from Protein Data Bank entry 1Y0F [40], [42]. The supramolecular structure in 1Y0F contains the Cα atomic coordinates of each amino acid as determined by low-resolution X-ray diffraction experiments [43]. The combined model had the helical propensity from the THeBuScr output and the supramolecular positions from the crystal structure. The linear telopeptides and side chain atoms were finally added using LeaP, part of the AMBER12 software package [44].

The triclinic unit cell dimensions came from the low resolution X-ray diffraction experiment of the collagen molecule [43], and are as described previously [39]. The system was solvated in LeaP by the addition of 11,980 explicit water molecules into the interstitial gaps between neighbouring collagen molecules. This value is equal to that derived by Streeter et al., with the aim of preserving the crystallographic dimensions of the fibril during an isothermal-isobaric MD simulation [39], whilst remaining in good agreement with experimental values for the intrafibrillar water content of 0.75 g water g^− 1^ of collagen [45]. All amino acids were assumed to be in their standard protonation states for physiological pH, resulting in 268 cationic sites from the amino acids with acidic side-chains and 235 anionic sites from the amino acids with basic side-chains. The remaining + 33 net charge was neutralised by 33 chloride ions per collagen molecule, resulting in an effective chloride concentration of 0.14 M, which is in general agreement with the experimentally observed concentration of 0.1 M sodium chloride [46], [47].

A single glucosepane cross-link was inserted into the collagen molecule between the residues identified during the distance-based criterion search (see below), totaling 24 unique models of collagen molecules with single cross-links. A native collagen model without cross-links but with an unbound D-glucose and minus four water molecules was also created to act as a reference for thermodynamic comparison to our covalently cross-linked collagen models.

### Distance-based criterion search

**Fig. 2:**
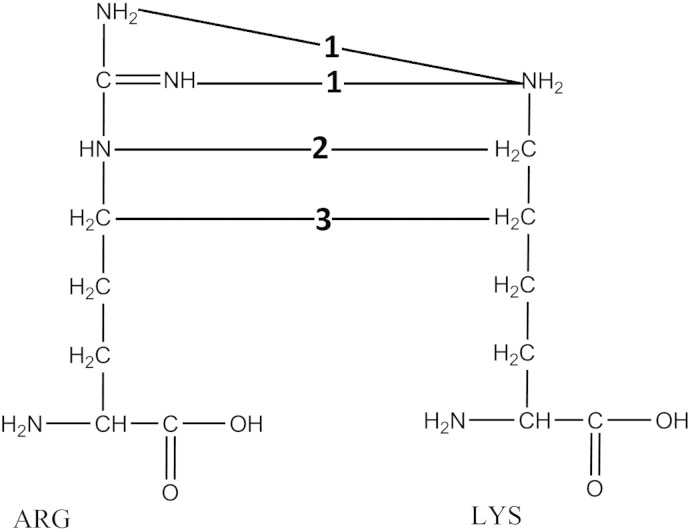
A schematic representation of the three points at which the distance was measured between the lysine and arginine during the distance based criterion search. Measurements are between: 1) arginine N^η^ and lysine N^ζ^, 2) arginine N^ε^ and lysine C^ε^, and 3) arginine C^δ^ and lysine C^δ^.

**Fig. 3:**
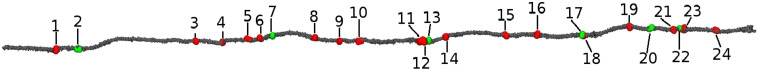
Distribution of the identified intra-molecular cross-linking sites along the length of the collagen molecule, red areas show energetically unfavourable sites and green areas show energetically favourable sites.

**Fig. 4:**
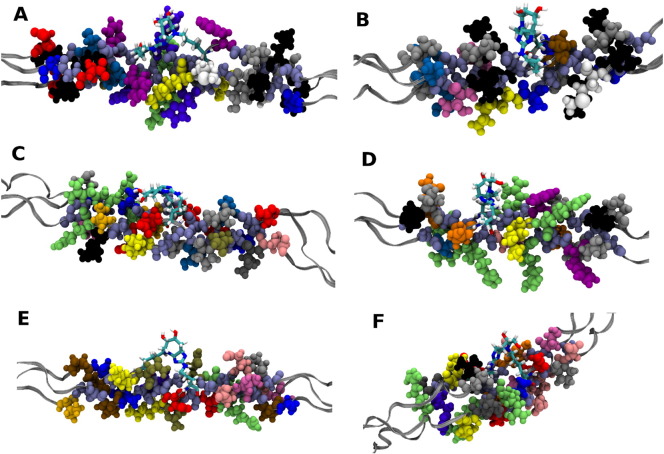
Local environment around the favourable glucosepane cross-link sites a) Position 2, b) Position 7, c) Position 13, d) Position 17, e) Position 20 and f) Position 22. (Residue colours: Ala — blue; Asn — tan; Asp — red; Arg — lime; Gln — orange; Glu — pink; Gly — ice blue; His — violet; Hyp — silver; Ile — grey; Leu — black; Lys — yellow; Met — white; Phe — purple; Pro — ochre; Ser — light blue; Thr — mauve; Tyr — magenta; Val — gold); glucosepane cross-link shown as sticks.

A custom script was used to scan the triple helical portion of a low energy conformer of a collagen molecule for lysine and arginine residues within a 5 Å cut-off across separate polypeptide chains, within the collagen molecule. The distance-based criterion of 5 Å was chosen based on two main factors. Firstly, previous studies have suggested that the two residues within proximity of 5 Å are a strong indicator for preferential glycation [21], and secondly, doubling the distance between the nitrogen atoms within glucosepane (approximately 2.5 Å and 3.7 Å) could reveal a reasonable number of potential sites where cross-links are likely to form [21]. The distance was calculated at three separate points along the residues' side chains, as shown in Fig. 2, namely between the three terminal nitrogen atoms lysine N^ζ^ and arginine N^η^; the lysine C^ε^ and the arginine N^ε^; and lysine C^δ^ and arginine C^δ^. Any site where at least one distance criterion was met was considered for cross-linking.

### Molecular dynamics simulations

MD simulations were performed on all models using SANDER, part of the AMBER12 software package [44]. Periodic boundary conditions were applied to the unit cell in order to simulate the densely packed fibrillar environment. The ff99SB force field was used for the parameterisation of the collagen molecule with additional terms based on published values for hydroxyproline [48]. Water molecules were represented using the TIP3P model [49]. The ff99SB force field was parameterized specifically for biological molecules and describes the non-bonded interactions by pairwise additive Lennard–Jones 6–12 potentials and pairwise additive coulombic potentials. Coulombic potentials were calculated using the Particle Mesh Ewald summation with a cut-off radius of 8.0 Å. A time step of 2 fs was adopted for all MD simulations and hydrogen-bond lengths were constrained using the SHAKE algorithm [50]. Constant temperature and pressure was maintained with the Berendsen algorithm [51] using a barostat time constant of 5.0 ps atm^− 1^ and a thermostat time constant of 1.0 ps. As the periodic unit cell has a *c* lattice parameter much larger than *a* and *b*, it is appropriate to use anisotropic coordinate rescaling rather than isotropic rescaling for maintaining constant pressure. This was achieved by making a small modification to the AMBER code, the details of which are discussed in our previous work [39]. To reduce instabilities in the starting structure, the models underwent steepest decent energy minimisation followed by a conjugate gradient minimisation. The system was heated to 310 K for 120 ps using the NVT ensemble followed by a further 320 ps using the NPT ensemble. Production simulations ran for 60 ns at 310 K using the NPT ensemble. Analysis was performed over the final 25 ns of the production simulations, as this was found in our previous work to give stable energies [39] and a fibrillar arrangement, which is in good agreement with experimental values [43]. The system density and the potential energy were monitored up to 35 ns at which point they were shown to converge.

## Figures and Tables

**Table 1:** The difference in enthalpy formation of all 24 identified intra-molecular cross-link sites. The six energetically favourable sites, shown here in bold, were aligned to ECM binding sites of the human collagen type I sequence. Column 1 gives the site number, columns two to four highlight the cross-linked residue pair between two of the three polypeptide chains (labelled using the UniProt residue number and the triple helical residue number shown in brackets) and the fifth column lists the change in enthalpy (kcal/mol).

Cross-link	Chain α1 (a)	Chain α1 (b)	Chain α2	ΔEnthalpy
1	^229^ARG(62)	^226^LYS(59)		–
**2**		^**257**^**ARG(90)**	^**183**^**LYS(87)**	**− 13.572**
3		^419^LYS(252)	^348^ARG(252)	+ 38.54
4	^458^ARG(291)		^386^LYS(290)	+ 7.883
5		^494^LYS(327)	^419^ARG(323)	+ 39.176
6	^509^LYS(342)		^438^ARG(342)	+ 4.357
**7**	^**527**^**LYS(360)**		^**456**^**ARG(360)**	**− 2.304**
8	^587^ARG(420)		^516^LYS(420)	+ 43.326
9	^620^ARG(453)		^549^LYS(453)	+ 76.636
10		^646^LYS(479)	^579^ARG(483)	+ 4.076
11	^734^ARG(567)	^731^LYS(564)		+ 23.157
12	^740^LYS(573)		^669^ARG(573)	+ 19.280
**13^α^**	^**748**^**LYS(581)**		^**677**^**ARG(581)**	**− 23.968**
14		^770^LYS(603)	^699^ARG(603)	+ 73.645
15	^854^ARG(687)	^851^LYS(684)		+ 92.728
16	^896^LYS(729)		^825^ARG(729)	+ 55.401
**17**	^**958**^**LYS(791)**	^**956**^**ARG(789)**		**− 2.315**
18	^958^LYS(791)		^884^ARG(788)	+ 65.516
19	^1025^ARG(858)	^1022^LYS(855)		+ 16.130
**20**	^**1055**^**ARG(888)**		^**980**^**LYS(884)**	**− 34.501**
21	^1085^LYS(918)	^1082^ARG(915)		+ 21.912
**22**	^**1094**^**ARG(927)**		^**1020**^**LYS(924)**	**− 36.130**
23	^1100^ARG(933)		^1029^LYS(933)	–
24	^1141^LYS(974)		^1073^ARG(977)	+ 90.852

**Table 2:** Biomolecule binding sites which overlap with the energetically favourable glucosepane cross-linking sites.

Cross-link	Aligned ECM binding sites	Enthalpy (kcal/mol)
**2**	Heat shock protein 47 [26], heparan sulphate [25], α1β1 integrin [24], α2β1 integrin [24], enzyme mediated mature cross-link [23]	− 13.572
**7**	Heat shock protein 47 [26], guanidine extracted decorin [22]	− 2.304
**13**	Phosphophoryn [27]	− 23.968
**17**	α2β1 integrin [24], heat shock protein 47 [26], Matrix Metalloproteinase 1 [28]	− 2.315
**20**	Dermatan sulphate [29], interleukin-2 [30]	− 34.501
**22**	Interleukin-2 [30], heparin [25], amyloid precursor protein [22]	− 36.130
